# Supplementary material for: A Portable Biodevice to Monitor Salivary Conductivity for the Rapid Assessment of Fluid Status
Source: J Pers Med. 2021 Jun 19;11(6):577. doi: 10.3390/jpm11060577 (PMC8235451; doi:10.3390/jpm11060577)
Supplement: Supplementary file 1 [file jpm-11-00577-s001.zip › jpm-1251504-supplementary.pdf]

Supplemental Material

Table S1. Different parameters for the evaluation of body fluid status in normal status.

Variables	Male (N=10)	Female (N=10)	P value
Salivary conductivity, μs/cm	3263.49±467.62	3262.79±1589.46	0.096
Serum osmolality, mOsm/kgH_2O	292.60±2.59	288.10±4.15	0.007**
Urine Osmolality, mOsm/kgH_2O	544.90 ±250.47	359.00 ±209.07	0.070
Urine SG	1.015 ±0.007	1.009 ±0.006	0.069
Thirst intensity CS	3.60±1.17	3.40±1.42	0.785
Thirst intensity VAS	3.75±1.89	3.82±2.34	0.821
Serum copeptin, pg/mL	198.89±78.08	244.25±99.03	0.496
FeNa, %	0.93±0.48	0.91±0.44	0.940

Note: Values are shown as the mean± standard deviation (SD). Abbreviations: FeNa: fractional excretion of sodium. Thirst intensity CS: thirst intensity categorical scale. Thirst intensity VAS: thirst intensity visual analog scale. Urine SG: urine specific gravity. Note: *P < 0.05, **P < 0.01, ***P<0.001.

Table S2. Different parameters for the evaluation of body fluid status in water restriction.

Variables	Male (N=10)	Female (N=10)	P value
Salivary conductivity, μs/cm	3867.96±1133.77	3474.93±1216.11	0.364
Serum osmolality, mOsm/kgH_2O	294.80±2.74	291.70±5.12	0.129
Urine Osmolality, mOsm/kgH_2O	831.10 ±150.86	744.50 ±239.83	0.450
Urine SG	1.024 ±0.004	1.021 ±0.006	0.239
Thirst intensity CS	5.60±0.70	6.00±0.47	0.118
Thirst intensity VAS	7.85±1.62	8.37±0.83	0.570
Serum copeptin, pg/mL	219.46±81.69	289.17±108.43	0.112
FeNa, %	0.37±0.18	0.40±0.23	0.940

Note: Values are shown as the mean± standard deviation (SD). Abbreviations: FeNa: fractional excretion of sodium. Thirst intensity CS: thirst intensity categorical scale. Thirst intensity VAS: thirst intensity visual analog scale. Urine SG: urine specific gravity. Note: *P < 0.05, **P < 0.01, ***P<0.001.

Table S3. Different parameters for the evaluation of body fluid status in rehydration status.

Variables	Male (N=10)	Female (N=10)	P value
Salivary conductivity, μs/cm	3201.82±897.66	2960.20±902.13	0.140
Serum osmolality, mOsm/kgH_2O	289.80±1.69	285.20±7.87	0.139
Urine Osmolality, mOsm/kgH_2O	110.40 ±69.04	128.00 ±130.89	0.880
Urine SG	1.003 ±0.002	1.004 ±0.004	0.691
Thirst intensity CS	2.30±1.64	1.90±0.99	0.749
Thirst intensity VAS	2.53±2.63	1.56±1.38	0.596
Serum copeptin, pg/mL	239.00±98.74	274.48±101.70	0.623
FeNa, %	0.63±0.23	0.48±0.10	0.112

Note: Values are shown as the mean± standard deviation (SD). Abbreviations: FeNa: fractional excretion of sodium. Thirst intensity CS: thirst intensity categorical scale. Thirst intensity VAS: thirst intensity visual analog scale. Urine SG: urine specific gravity. Note: *P < 0.05, **P < 0.01, ***P<0.001.
